# Hydrogen sulfide alleviates pericarp browning in lichi fruit by modulating energy and sugar metabolisms

**DOI:** 10.3389/fpls.2024.1421203

**Published:** 2024-09-03

**Authors:** Zhaoyin Gao, Kunkun Zhao, Zhengke Zhang, Mir Muhammad Nizamani, Songgang Li, Min Li, Deqiang Gong, Jiabao Wang, Meijiao Hu

**Affiliations:** ^1^ Tropical Crops Genetic Resources Institute, Chinese Academy of Tropical Agricultural Sciences, Haikou, China; ^2^ School of Food Science and Engineering, Hainan University, Haikou, China; ^3^ Department of Plant Pathology, Agricultural College, Guizhou University, Guiyang, China; ^4^ Environment and Plant Protection Institute, Chinese Academy of Tropical Agricultural Sciences, Haikou, China

**Keywords:** litchi, browning, hydrogen sulfide, energy, sugars

## Abstract

Postharvest litchi is susceptible to browning that limits the development of litchi industry. Hydrogen sulfide (H_2_S) is an important bioactive molecule that can regulate many physiological processes. This study examined the effects of exogenous H_2_S on pericarp browning and related physiological mechanisms in postharvest litchi. The results exhibited that exogenous H_2_S treatment delayed the browning of litchi pericarp and reduced the damage to cell membrane integrity during storage. This treatment inhibited the energy losses of litchi fruit by increasing the activities of H^+^-ATPase, Ca^2+^- ATPase, cytochrome C oxidase (CCO) and succinate dehydrogenase (SDH) and regulating the expression of energy metabolism-related genes, including *LcAtpB*, *LcSnRK2*, *LcAAC1*, *LcAOX1* and *LcUCP1*. In addition, H_2_S treatment increased the levels of fructose, glucose, sucrose, inositol, galactose and sorbose in litchi fruit, and promoted sucrose synthesis by regulating the activities of sucrose phosphate synthase (SPS), sucrose synthase (SS), acid invertase (AI) and neutral invertase (NI). Based on the current findings, we suggest that exogenous H_2_S enhances the energy supply and antioxidant activity of litchi by modulating energy and sugar metabolism, thereby inhibiting fruit browning and senescence. These results indicated that H_2_S treatment is an effective approach to maintaining the quality of litchi fruit and extending its shelf life.

## 1 Introduction

Litchi (*Litchi chinensis* Sonn.) is a typical tropical fruit crop with high market acceptance because of its attractive red skin, sweet taste and rich nutrition (Pareek, 2016). However, the rapid pericarp browning after harvest seriously restricts the expansion of the litchi industry (Jiang et al., 2006). Fruit senescence is a programmed process involving multiple complex factors (Figueroa et al., 2021). Previous studies have reported that the oxidative damage to cell membrane is a key factor in causing litchi senescence and browning (Jiang et al., 2004; Deshi et al., 2020). Adenosine triphosphate (ATP) is a cellular “energy currency”, whose sufficient supply can enhance the antioxidant activity in cells and maintain normal physiological functions of cell membrane and cell wall in plants (Aghdam et al., 2018). A variety of researches have been confirmed that delay of fruit senescence and quality deterioration by various postharvest treatments could be involved in the improvement of energy state via regulation of energy metabolic enzymes and related genes, as demonstrated in litchi, longan, mango and pear fruits (Zhang et al., 2017; Wang et al., 2020a; Zhang et al., 2023a; Huang et al., 2024).

Sucrose, glucose and fructose are the foundational sources of energy in plants. They are not only responsible for the energy supply of fruit but also determine the flavor and nutritional value (Fan et al., 2021). Moreover, soluble sugars, as an important signaling molecules in plants, are also considered to be associated with abiotic stress responses, which are involved in osmotic adjustment and activation of antioxidant system (Saddhe et al., 2021). Some reports suggest that higher contents of sucrose, fructose, glucose due to 24-epibrassinolide and fibroin treatments could contribute to the improvement of energy state and stress resistance of fruit, hence aiding in the prevention of postharvest senescence in peach and banana fruit (Liu et al., 2019; Hu et al., 2023). Song et al. (2016) noted that chitosan/nano-silica treatment resulted in more accumulation of glucose and fructose in loquat in the low-temperature environment, which promoted the increase of antioxidant activity, reduced membrane damage, alleviating chilling injury of loquat fruit. For litchi, our recent research has confirmed that the response of litchi fruit to energy deficiency was involved in the modulation of sugar metabolic pathways (Zhao et al., 2024). However, the role of sugars in maintaining the quality of litchi fruit still requires further investigation.

Hydrogen sulfide (H_2_S), the third identified gaseous signaling molecule after hydrogen peroxide (H_2_O_2_) and nitric oxide (NO) in plants, plays undeniable roles in growth and development and response to environmental stresses (Arif et al., 2021). Emerging evidence indicates that H_2_S treatment at low concentration (μM) elicits beneficial effects on storability in postharvest crops. For example, exogenous H_2_S treatment (500 μM and 0.735 μM) was deemed to be capable of delaying senescence and improving stress resistance in postharvest fruits of banana and navel orange through enhancing cellular energy state, augmenting antioxidant capacity or regulating phenylpropanoid metabolism pathway (Li et al., 2016; Huang et al., 2023b). Moreover, H_2_S treatment, as a potential postharvest preservation technique, is attracting attention due to its straightforward operation, low cost, and the capability to be readily applied to large-scale fruit processing (Alhassan et al., 2022). Recent studies indicated that H_2_S treatment enhanced the activities of antioxidant enzymes in litchi, which led to reduced oxidative damage to the cell membrane and improved the fruit quality during storage (Deshi et al., 2020; Siddiqui et al., 2021). However, the influence of H_2_S treatment on postharvest litchi browning via energy and sugar metabolisms and its possible mechanism remain unclear. Therefore, this study aimed to elucidate the effects of exogenous H_2_S on postharvest browning of litchi in relation to membrane integrity, energy state, energy metabolism-related enzyme activities and gene expression, sugars content, and sucrose metabolism-related enzyme activities. The results might offer fresh insights into regulatory mechanisms of H_2_S effecting postharvest browning in litchi fruit.

## 2 Materials and methods

### 2.1 Fruit material and treatment

Litchi fruit (*L. chinensis* Sonn. cv. ‘A4 Wuhe’) with a commercial maturity were harvested in the litchi garden situated in Yongxing Town within Haikou, China. The picked fruit was transported to the laboratory within 2 hours. The selection criteria of litchi fruit for experiment were uniform size, color, and shape, without pericarp damage or disease symptoms. All fruit were treated with 0.1% (v/v) prochloraz for 3 min, washed with deionized water, then were randomly divided into two groups (576 fruit/group) after drying. The two groups of fruit were soaked in distilled water (control group) and 1.0 mM sodium hydrosulfide (H_2_S donor, experimental group) for 15 min, respectively. After drying, all fruit were placed in plastic boxes (without plastic covers), each group contained 48 boxes (12 fruit per box), then stored at 25 ± 1°C and 85 ± 5% relative humidity. During storage, samples were collected at 0, 12, 24, 48, 72, and 96 h. During each sampling opportunity, eight boxes were randomly selected from both the experimental and control groups, respectively, for the physiological investigation of fresh samples. This included the assessments of browning index (BI; 30 fruit defined as one replicate), respiration rate (20 fruit defined as one replicate) and membrane permeability (6 fruit defined as one replicate), with three replications for each parameter. Furthermore, pericarp samples from six litchi fruit were taken at each sampling point, frozen in liquid N_2_ and stored at -80°C for subsequent analysis. Each parameter underwent three measurements.

### 2.2 BI, respiration rate, membrane permeability, and malonaldehyde content

Pericarp browning was categorized into five scales depending on the proportion of browned area on the litchi surface (Wang et al., 2020a). 0, no browning; 1, ≤ 1/4 browning; 2, 1/4 - 1/2 browning; 3, 1/2 - 3/4 browning; 4, ≥ 3/4 browning. The BI was calculated as follows: BI = ∑(browning scale × number of fruit per class)/(total number of fruit per replicate of each treatment × maximum browning scale).

Respiration rate was assessed following the protocol outlined by Li et al. (2019). The litchi fruit were placed in a 4-L plastic containers for 20 min at 20°C, then obtaining the CO_2_ concentration using an infrared CO_2_ analyzer (CXH-3010E, Beijing, China). Respiration rate is the quantity of carbon dioxide generated per kilogram fresh weight (FW) every second (µmol kg^−1^ s^−1^).

The membrane permeability was reflected by relative electrolyte leakage (Zhang et al., 2017). Thirty discs of the pericarp were meticulously procured from each set of six fruit in every experimental replication, with a cork borer (8 mm) as the extraction tool. The discs were cleaned twice and incubated in 50 mL deionized water at 25°C for 30 min. The solution’s initial electrolyte conductivity (Ei) was detected using a conductivity meter. The total electrolyte value (Et) was determined after boiling the solution with discs for 20 min and then cooling it to 25°C. Relative electrolyte leakage was calculated as follows:


Relative electrolyte leakage (%) = Ei/Et × 100%

MDA content was analyzed using 3 g pericarp tissues following the procedure outlined by Li et al. (2019). MDA content was defined as µmol kg^−1^ FW.

### 2.3 Measurements of adenosine triphosphate, adenosine diphosphate (ADP), adenosine monophosphate (AMP) content and calculation of energy charge (EC)

The contents of ATP, ADP, and AMP in litchi pericarp were determined using the methodology outlined by Zhang et al. (2017).


EC = (ATP + 1/2ADP)/(ATP + ADP + AMP)

### 2.4 Enzymes activities related to energy metabolism

The activities of H^+^-ATPase, Ca^2+^-ATPase, cytochrome C oxidase (CCO) and succinate dehydrogenase (SDH) were assessed using the procedures outlined in Jin et al. (2013). Enzyme activities were quantified as U kg^-1^.

### 2.5 Determination of gene expression levels

Genes’ expression levels were assessed using real-time quantitative PCR (RT-qPCR). 1.5 g pericarp tissues was taken and used for the extract-purifying total RNA using Quick RNA isolation Kit (0416-50 GK; Huayueyang Biotech, Beijing, China). The cDNA fragment was obtained by reverse transcription through FastQuant RT Kit (KR106; Tiangen Biotech, Beijing, China) using the purified RNA template. The RT-qPCR primer sequences of *LcAtpB* (ATP synthase β-subunit), *LcAOX1* (alternative oxidase 1), *LcUCP1* (mitochondrial uncoupling protein 1), *LcAAC1* (ADP/ATP carrier 1), and *LcSnRK2* (sucrose non-fermenting-1-related kinase 2) were referred to the report of Wang et al. (2013). Fluorescence quantitative Kit uses SYBR^®^ Premix^Ex^ Taq™ (Tli RNaseH Plus; Takara Biotech, Dalian, China). RT-PCR uses Life QuantStudio6 Flex with a 10 µL PCR reaction system (0.3 μL forward primer; 0.3 μL reverse primer; 0.2 μL ROX Reference Dye II; 1.0 μL cDNA; 8.2 μL ddH_2_O). The *LcActin* gene (GenBank ID: DQ990337.1) was employed as a reference for quantitative standardization during amplification. Gene expression levels were quantified applying the 2 ^−△△CT^ method.

### 2.6 Components and contents of sugars

Sugars were obtained and determined utilizing the protocol of Wu et al. (2016) with some changes. The litchi pericarp of 0.2 g was cooked in a microwave at 100°C for 30 s to disable the enzyme. The sample was homogenized in a mortar, extracted thrice with ethanol, and then diluted to 9 mL. The mixture was centrifuged at 13000 g for 15 min. The extraction solution was diluted 5-fold with deionized water. The 0.5 ml extraction solution was transferred into a 1.5 ml centrifuge tube and blow dry with nitrogen. A solution of 30 µL methylammonium chloride in pyridine (20 g L^−1^) was poured into the centrifuge tube and shaken at 650 rpm for 1.5 h at 37°C. The N, O-bis (trimethylsilyl) trifluoroacetamide with 1% trimethylchlorosilane (70 µL) was added and shaken at 650 rpm for one hour at 70°C. The supernatant (1 µL) was passed through the Agilent HP-5MS (30 m × 0.25 mm × 0.25 µm) after standing for 30 min at ambient temperature. The sugars in litchi were detected by gas chromatography using Agilent 7894A-5975C GC-MS (Agilent Technology, Palo Alto, CA, USA). An external standard solution was utilized to calculate the sugar concentration in litchi.

### 2.7 Activities of sucrose metabolic enzymes

The enzymatic activities of sucrose phosphate synthase (SPS), sucrose synthase synthesis (SS-s), sucrose synthase cleavage (SS-c), acid invertase (AI) and neutral invertase (NI) were evaluated using the methodologies specified in Sun et al. (2020). The enzymatic activity for SS-c, AI, and NI was ascertained based on the volume of enzyme that could yield 1 mg reducing sugar within a minute, with the absorbance for SS-c measured at 540 nm, and for AI and NI, at 510 nm. Conversely, the activity of SS-s and SPS was gauged by the amount required to synthesize 1 mg sucrose per minute, utilizing a wavelength of 480 nm for these assays. Enzyme activity were quantified as U kg^-1^.

### 2.8 Statistical analysis

The data is displayed as the mean ± standard error (SE). T-test analysis was conducted to compare the difference between control and experimental groups at the same day (*P < 0.05, **P < 0.01), using SPSS 27.0.1.

## 3 Results

### 3.1 Pericarp browning, respiration rate, membrane permeability, and MDA content

**Figure 1:**
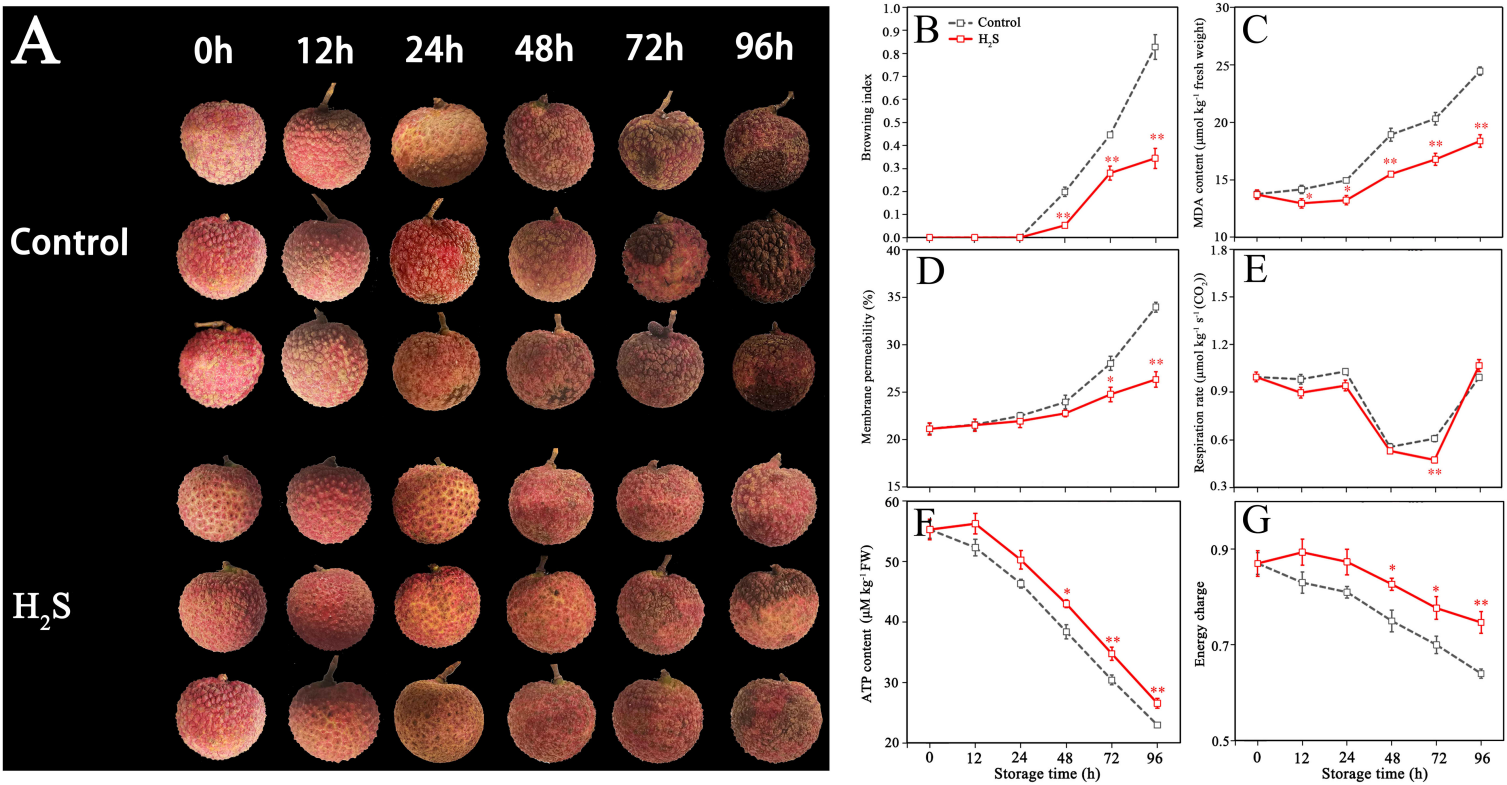
Appearance **(A)** of control and H_2_S treated fruit during storage, as well as corresponding changes in browning index (BI) **(B)**, malonaldehyde (MDA) content **(C)**, membrane permeability **(D)**, respiration rate **(E)**, adenosine triphosphate (ATP) **(F)**, and energy charge (EC) **(G)**. Data are the mean ± standard error (n=3). The asterisk represents the significant difference (* P < 0.05, ** P < 0.01) between the experimental and control groups.

The control fruit did not show browning symptoms in first 24 h of storage, the BI sharply increased after 24 h and reached 0.83 ± 0.05 at 96 h of storage (**Figures 1A, B**). H_2_S treatment effectively delayed pericarp browning, in which the BI was still at a low level (0.34 ± 0.04) after storage for 96 h.

The MDA content in control fruit increased continuously during storage with initial 13.8 ± 0.6 to final 24.5 ± 0.7 µmol kg^−1^ (**Figure 1C**). The MDA content of litchi pericarp was consistently 15% lower on average for the entire storage period due to H_2_S treatment, indicating that H_2_S treatment inhibited the oxidative damage to cell membrane.

**Figure 1D** illustrates that the relative electrolyte leakage in the control group gradually increased from 21.13% to 33.96% during storage. Relative to the untreated specimens, the average values of relative electrolyte leakage for fruit treated with H_2_S exhibited a reduction of 12% during 24 to 96 h, indicating that H_2_S treatment effectively maintained the integrity and functionality of the cell membrane.

**Figure 1E** illustrates that the respiration rate of control fruit remained relatively stable within the first 24 h, decreased rapidly in 24-48 h, then increased sharply in 72-96 h. The respiration rate of fruit treated with H_2_S was inhibited during storage for 0-72 h.

### 3.2 Energy state

As shown in **Figures 1F, G**, ATP content and EC in litchi pericarp decreased sharply as storage time increased in the control fruit. H_2_S treatment prevented the reduction in ATP content and EC, with significant variations observed between 48 to 96 h.

### 3.3 Enzymatic activity involved in energy metabolism

**Figure 2:**
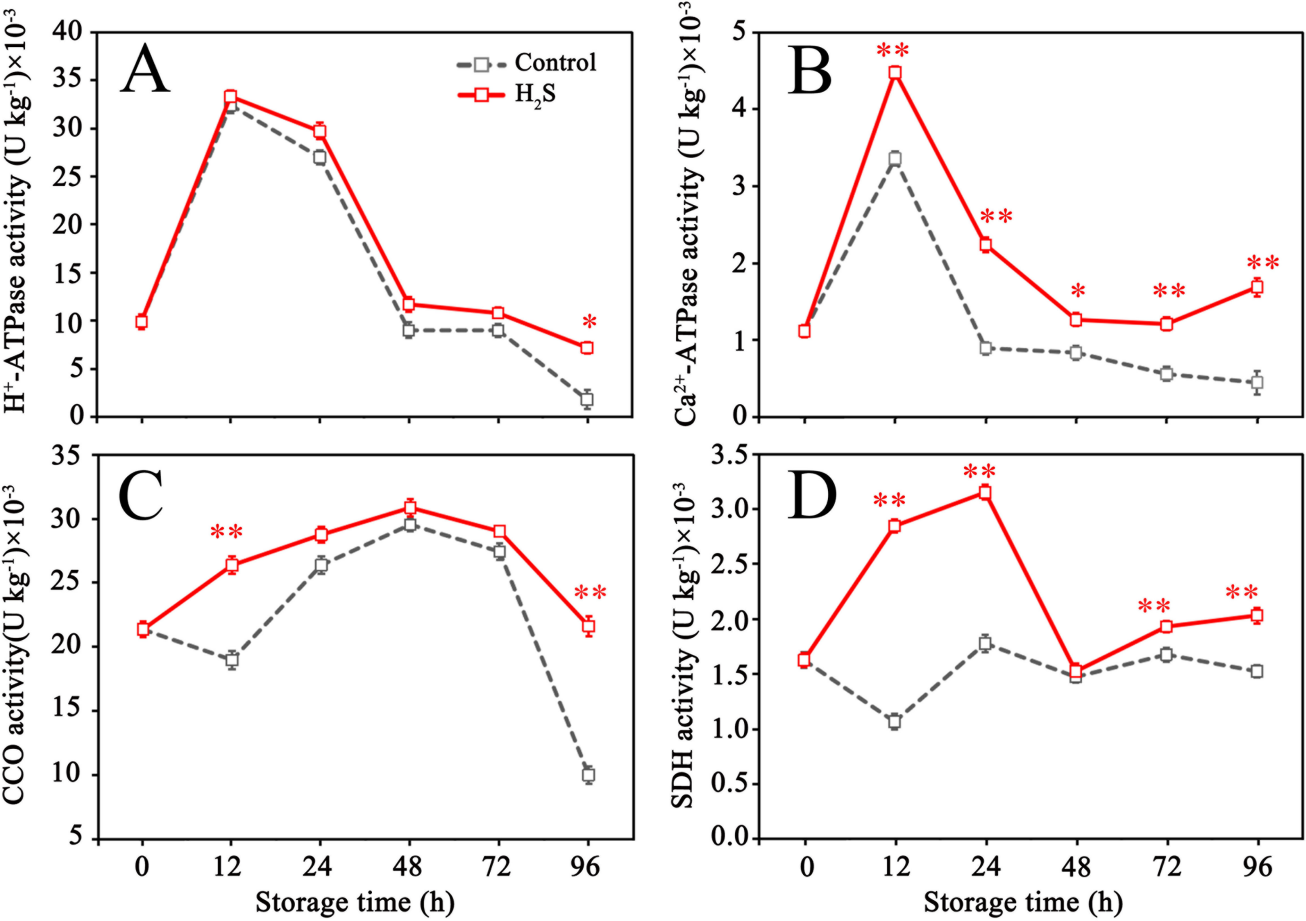
H^+^-ATPase **(A)**, Ca^2+^-ATPase **(B)**, cytochrome C oxidase (CCO) **(C)** and succinate dehydrogenase (SDH) **(D)** activities in control and H_2_S treated fruit during storage. Data are the mean ± standard error (n=3). The asterisk represents the significant difference (* P < 0.05, ** P < 0.01) between the experimental and control groups.

**Figures 2A, B** showed that the activities of both H^+^- ATPase and Ca^2+^-ATPase initially increased and subsequently gradually declined over the storage period in the control group. CCO activity reduced somewhat from 0 to 12 hours, then gradually climbed, reaching its peak at 48 hours before decreasing (**Figure 2C**). SDH activity exhibited a change with fluctuation during storage (**Figure 2D**). Generally, H_2_S treatment increased the activities of H^+^- ATPase, Ca^2+^-ATPase, CCO and SDH during storage (**Figures 2A–D**), but its effects on Ca^2+^-ATPase and SDH were more profound (**Figures 2B, D**).

### 3.4 Expression of the energy metabolism-related gene

**Figure 3:**
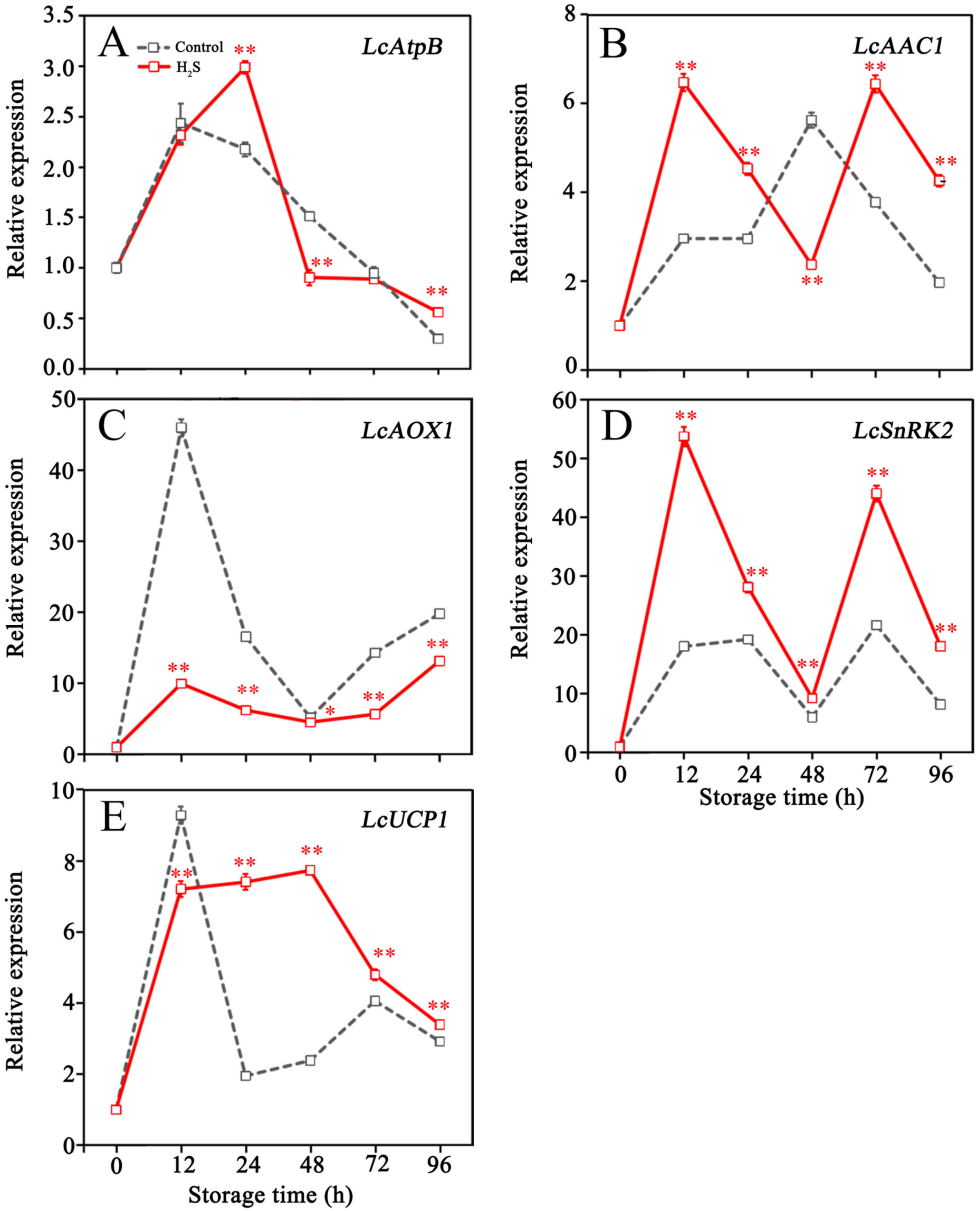
Relative expression of LcAtpB **(A)**, LcAAC1 **(B)**, LcAOX1 **(C)**, LcSnRK2 **(D)** and LcUCP1 **(E)** in control and H_2_S treated litchi fruit during storage. Data are the mean ± standard error (n=3). The asterisk represents the significant difference (* P < 0.05, ** P < 0.01) between the experimental and control groups.

**Figure 3** displayed the effects of H_2_S treatment on the expression of genes associated with energy metabolism pathways. Transcript abundances of *LcAtpB*, *LcAOX1* and *LcUCP1* in control fruit peaked at 12 hours and then experienced an overall decline throughout the remainder of the storage period (**Figures 3A, C, E**). H_2_S treatment remarkably enhanced *LcAtpB* expression at 24 h, promoted *LcUCP1* expression within 24 to 96 h, and inhibited *LcAOX1* expression during storage (**Figures 3A, C, E**). Expression of *LcAAC1* in control fruit increased by 5.6-fold within 48 hours, then declined until the end of storage (**Figure 3B**). H_2_S treatment promoted *LcAAC1* expression during storage, except for the value at 48 h (**Figure 3B**). *LcSnRK2* expression in control fruit displayed a fluctuating changes, increased at 24 h and 72 h, but decreased at 48 h and 96 h (**Figure 3D**). Comparatively, higher levels of *LcSnRK2* expression were detected in the litchi fruit treated with H_2_S during storage, especially at 12 h and 72 h (**Figure 3D**).

### 3.5 Components and content of sugars

**Figure 4:**
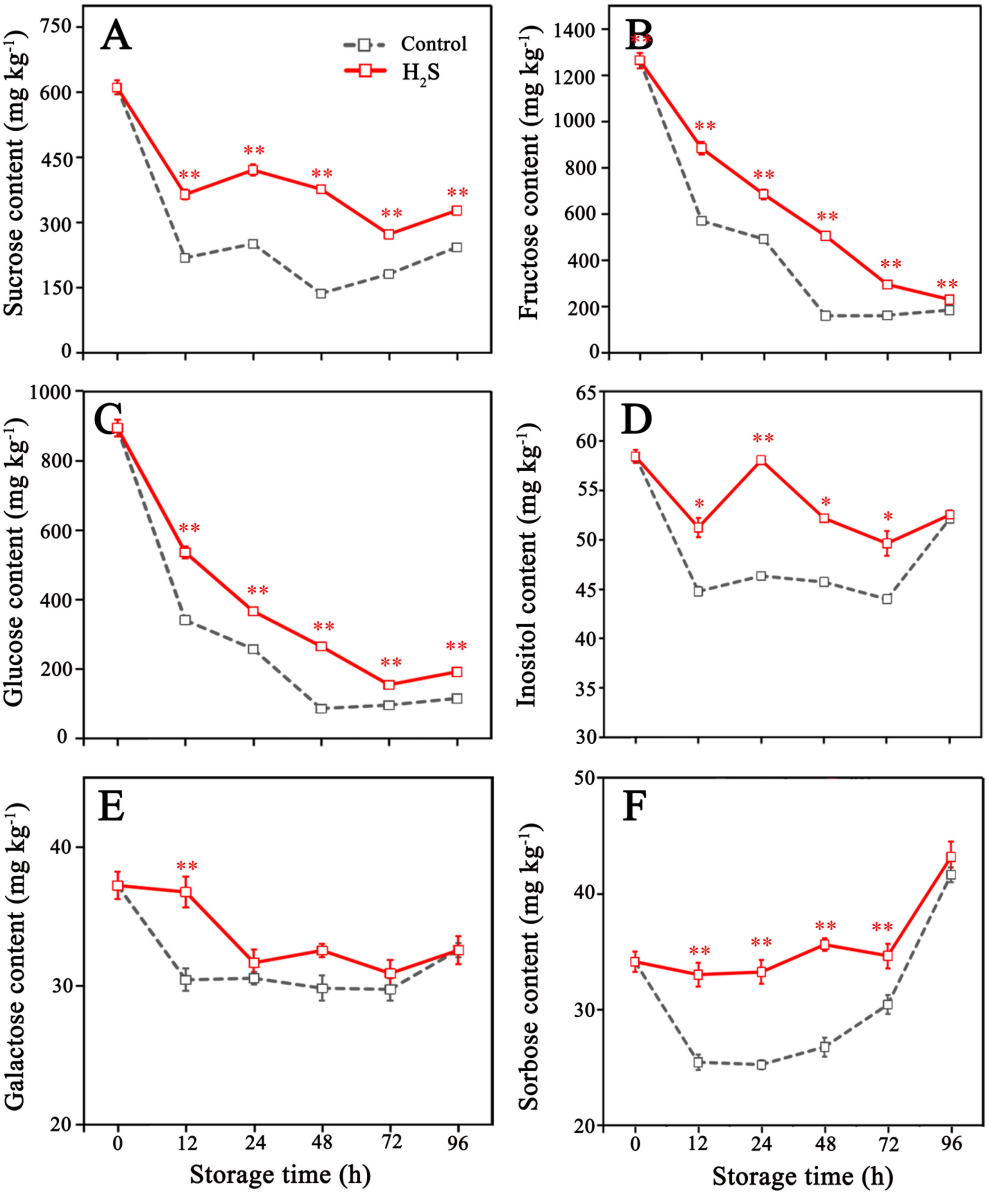
Contents of sucrose **(A)**, fructose **(B)**, glucose **(C)**, inositol **(D)**, galactose **(E)** and sorbose **(F)** in control and H_2_S treated litchi fruit during storage. Data are the mean ± standard error (n=3). The asterisk represents the significant difference (* *P* < 0.05, ** *P* < 0.01) between the experimental and control groups.

In the litchi pericarp, six types of sugars were detected, with initial concentrations of 1263 mg kg^−1^ (fructose), 894 mg kg^−1^ (glucose), 611 mg kg^−1^ (sucrose), 58 mg kg^−1^ (inositol), 37 mg kg^−1^ (galactose), and 34 mg kg^−1^ (sorbose), respectively (**Figure 4**). The content of fructose, glucose and sucrose in control fruit rapidly decreased in the early stage of storage and reached the minimum at 48 h, followed by slight increases until the end of storage (**Figures 4A–C**). The H_2_S treatment delayed decrease in fructose, glucose and sucrose content, which reached the minimum after 72 h. The contents of inositol, galactose, and sorbose in litchi fruit of control were low and remained relatively stable during storage. However, a notable increase in the concentrations of these sugars was observed in fruit subjected to H_2_S treatment when contrasted with those that received no treatment (**Figures 4D–F**).

### 3.6 Enzymatic activity involved in sucrose metabolism

**Figure 5:**
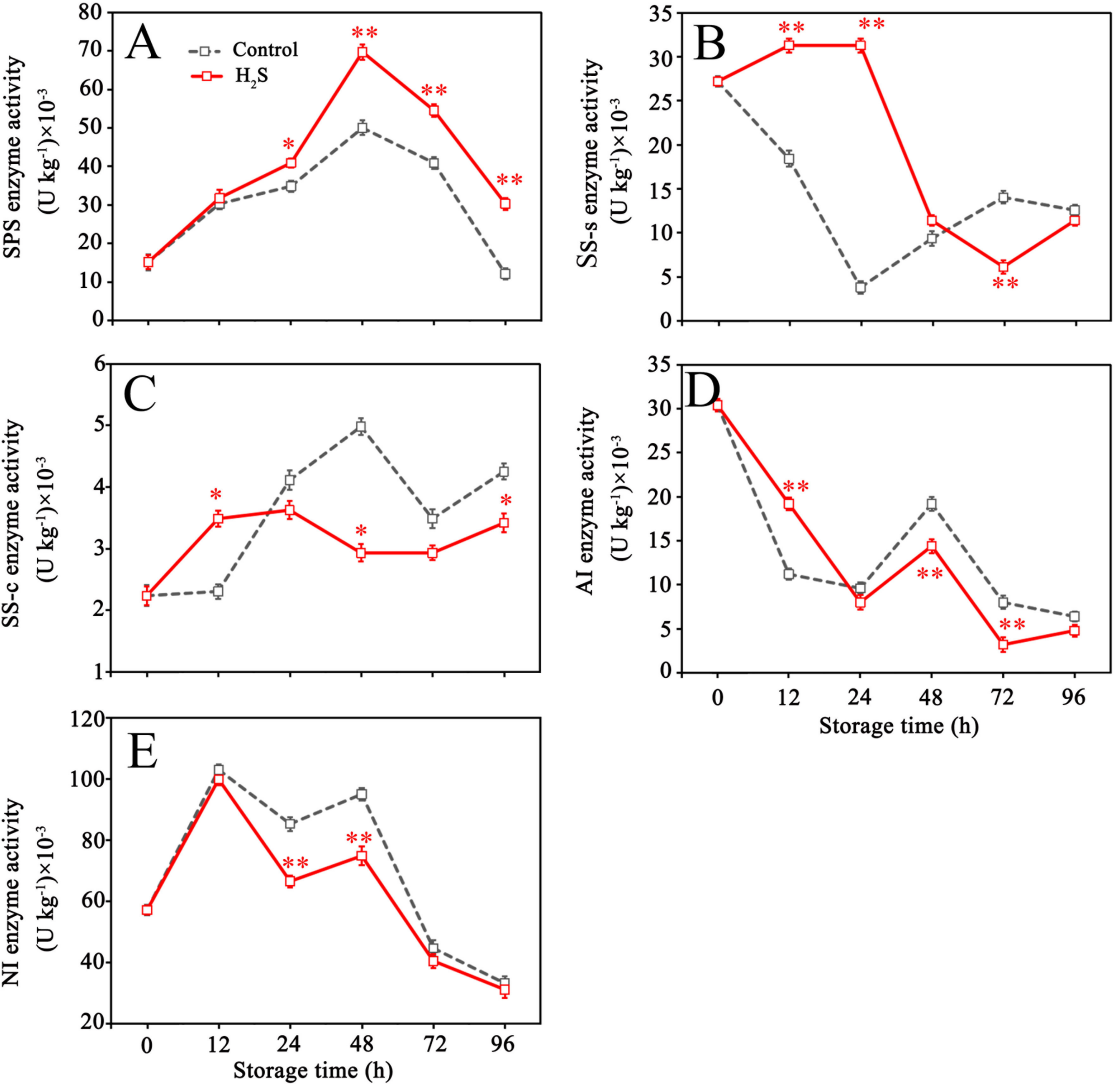
Sucrose phosphate synthase (SPS) **(A)**, sucrose synthase synthesis (SS-s) **(B)**, sucrose synthase cleavage (SS-c) **(C)**, acid invertase (AI) **(D)** and neutral invertase (NI) **(E)** activities in control and H_2_S treated fruit during storage. Data are the mean ± standard error (n=3). The asterisk represents the significant difference (* P < 0.05, ** P < 0.01) between the experimental and control groups.

**Figure 5** illustrates that the SPS activity in control fruit rose during the initial phase of storage, peaked at 48 h, and thereafter declined. AI activity in control fruit decreased within 0-24 h, then increased within 24-48 h and dropped after 48 h. SS-s activity declined within 0-24 h, then increased within 24-72 h and decreased again after 72 h. Contrary to the change of SS-s activity, SS-c activity climbed from 0 to 48 h, reduced within 48-72 h, then increased again. The activity of SS-s was higher than that of SS-c at the same period, as seen in **Figures 5B, C**. NI activity decreased after reaching the peak at 12 h, then continued to decrease after slightly increasing from 24 to 48 h. H_2_S treatment enhanced the activities of SPS and SS-s (0-48 h), while reduced the activities of SS-c (24-96 h), AI (24-96 h) and NI during storage.

## 4 Discussion

### 4.1 Effect of H_2_S on pericarp browning in litchi in relation to energy state

Rapid pericarp browning in harvested litchi fruit significantly reduces shelf life and compromises fruit quality, which limits the development of the litchi industry (Huang et al., 2023a). Recently, many alternative methods have been emerged to alleviate litchi browning, including radiation processing, modified atmosphere packaging, edible coating, organic acid treatment, and plant extracts treatment (Zhang et al., 2023a). In the present study, we observed that the browning symptoms of litchi were alleviated following treatment with H_2_S (**Figure 1A**). The results were consistent with the reports on H_2_S delaying quality deterioration in apples (Chen et al., 2021), bananas (Ge et al., 2017) and grapes (Ni et al., 2016).

During storage of fruit, the reactive oxygen species (ROS) in cells constantly accumulate, which leads to oxidative damage of biomacromolecules (e.g., DNA, proteins and lipids), destroy the function of membrane systems and organelles, thereby triggering browning and senescence in fruit pericarp (Chen and Yang, 2020; Xu et al., 2023). When sufficient energy is available in fruit, the activities of enzymatic/non-enzymatic antioxidant systems can be maintained, contributing to inhibition of ROS accumulation, maintenance of cell membrane integrity and delayed browning in litchi fruit (Yi et al., 2010). Therefore, ensuring the energy requirements of cells is of paramount importance for safeguarding the normal physiological functions of the fruit and improving its quality (Hu et al., 2023). The research on H_2_S functions has confirmed that endogenous H_2_S as a regulatory factor in the energy metabolism of eukaryotes, can facilitate the cell’s energy production under adverse conditions and fulfill the demands for normal growth and development (Fu et al., 2012). Additionally, research has shown that H_2_S treatment actively contributes to reducing oxidative damage to cell membranes and enhancing the overall antioxidant capacity of litchi fruit (Deshi et al., 2020). According to the outcomes of experiments, H_2_S treatment maintained a higher energy state of litchi fruit and alleviated the membrane integrity damage, as reflected by the enhancement of ATP and EC levels, and the reduction of MDA content and membrane permeability. The data above suggest that H_2_S-delayed litchi pericarp browning may be associated with the improvement of energy state, which is consistent with the report of He et al. (2022).

### 4.2 Effect of H_2_S on activities of energy metabolic enzymes

Previous research studies demonstrated that energy state is associated with the activities of energy metabolism-related enzyme, mainly involving H^+^-ATPase, Ca^2+^-ATPase, CCO and SDH (Ali et al., 2020). H^+^-ATPase and Ca^2+^-ATPase can catalyze ATP decomposition to release energy and form ADP and free phosphate ion (Wang et al., 2020a). SDH and CCO are both key enzymes in the tricarboxylic acid (TCA) cycle and electron transport chain (ETC). SDH facilitates the conversion of succinate to fumarate in TCA cycle, while promoting the reduction of ubiquinone to ubiquinol in the ETC. CCO as a terminal oxidase in the ETC, provides energy for cells through coupling oxidative phosphorylation (Wang et al., 2020a). Aghdam et al. (2018) found that the reduction in the activities of energy metabolism-related enzymes had a negative effect on mitochondrial function, which could worsen fruit quality. For example, the reduction in H^+^-ATPase activity disrupts cellular ionic and pH homeostasis, exacerbating the senescence process of fruits (Zhang et al., 2017). Similarly, the inhibition of Ca^2+^-ATPase activity increases the Ca^2+^ accumulation, which is also closely associated with the senescence process of fruits (Zhang et al., 2017). Furthermore, CCO and SDH play active roles in energy production (Li et al., 2016). Their activities directly influence the energy state of fruits and are crucial for maintaining their metabolic vitality. The results showed that H_2_S treatment increased the activities of H^+^-ATPase, Ca^2+^-ATPase, CCO and SDH during litchi storage, indicating that H_2_S might improve energy state by upregulating the catalytic activity of energy-related enzymes, thereby inhibiting pericarp browning. Consistent with this finding, H_2_S application to bananas significantly enhanced the activities of H^+^-ATPase, Ca^2+^-ATPase, CCO and SDH, therefore maintaining the energy charge, which helped to improve fruit quality and extend the shelf life of fruit (Li et al., 2016).

### 4.3 Effect of H_2_S on the expression of energy metabolism-related genes

AtpB is one of the core components in F_1_Fo-ATP synthase and the function significantly influences the process of oxidative phosphorylation in respiration pathway (Brandt et al., 2013). In addition, the role of AtpB extends beyond energy production, it is also identified as a protein that induces apoptosis in response to various stress signals, can trigger the cell programmed death (Chivasa et al., 2011). In the context of pericarp browning, litchi fruit senescence has been associated with a fast increase in the expression of the AtpB gene, as documented by Liu et al. (2015). The results in this study revealed that the application of H_2_S can delay the surge in *LcAtpB* expression while reducing the browning index, suggesting that ability of H_2_S prolonging the shelf life of litchi may be attributed to its regulation on *LcAtpB* expression.

As a cellular energy sensor, SnRK can perceive energy state of the cell, and promote energy production and reduce energy consumption when energy shortage occurs (Nietzsche et al., 2014). Meanwhile, the activation of SnRK2 is thought to be in concert with the expression of genes involved in energy metabolism, which may cause an increase in ATP synthesis and a delay in fruit senescence (Aghdam et al., 2018). The results showed that the *LcSnRK2* expression was upregulated in litchi during early storage, which might be a response to the ATP deficiency; this is consistent with findings by Zhang et al. (2017). The *LcSnRK2* expression was enhanced following H_2_S treatment, suggesting that this treatment could modulate the energy supply of litchi by influencing the expression of *LcSnRK2*.

As a vital mitochondrial carrier protein, the primary function of AAC is to facilitate the exchange of ADP and ATP and meet the energy needs of the cell (Klingenberg, 2008). Similar to the function of AtpB, AAC also plays a significant role in the process of programmed cell death (Huang et al., 2014). The research reveals that the function dysfunction of AAC can lead to various diseases, which are related to the disruption of mitochondrial energy production (Clémençon et al., 2013). In the results of this study, H_2_S treatment upregulated the *LcAAC1* expression, which might help maintain the energy state of litchi fruit and contribute to delayed pericarp browning.

AOX and UCP are energy dissipation systems that are universally present in plant mitochondria (Pu et al., 2015). In the electron transfer chain, AOX can receive electrons and transfer them to oxygen, bypassing complexes III and IV, and reducing EC levels, while UCP can suppress ATP production through dissipating the proton electrochemical gradient produced by the ETC (Pu et al., 2015). Both AOX and UCP serve crucial roles in defending cells from oxidative damage and maintaining energy balance (Borecký et al., 2006). For example, AOX and UCP have been shown to regulate the energy state and ethylene release during the senescence of papaya fruit (Oliveira et al., 2015). Li et al. (2020a) found that reducing the expression of certain AOX and UCP genes can extend the shelf life of longan fruit, which was attributed to decrease in the energy dissipation. In this research, the expression of *LcAOX1* and *LcUCP1* in the control litchi fruit decreased overall after 12 h, indicating that the function of energy dissipation system could be inhibited when energy state declined. In contrast to the effects on *LcAOX1* expression, H_2_S treatment enhanced the *LcUCP1* expression during litchi storage for 24-96 h. In fruit, the functions of UCP and AOX have been demonstrated to be complementary, with the expression levels being jointly regulated by external stress and cellular signaling (Li et al., 2020a). The upregulation of *LcUCP1* expression suggested that it might participate in regulating the fruit’s energy balance and mitigate oxidative damage as the major energy dissipation system when energy state in litchi improved in response to H_2_S.

### 4.4 Effect of H_2_S on the sugar metabolism

The content of soluble sugars is a key parameter for assessing fruit quality, not only determining the sweetness of fruit but also having a significant impact on the energy provision, cellular matrix stability, and stress tolerance (Wang et al., 2020b). In litchi fruit, the exceptional sweetness is a direct result of high sugar content, which accounts for 15–20% of the fresh weight, primarily consisting of sucrose, fructose, and glucose (Wang et al., 2006). In the present study, the application of H_2_S effectively preserved the levels of six sugars in the litchi fruit during postharvest storage, while reducing the respiration rate. Previous research indicated that H_2_S treatment could improve the ratio of pentose phosphate pathway in fruit, while reducing the Embden-Meyerhof-Parnas-tricarboxylic acid cycle in respiratory pathways. This regulation reduced the respiration rate and the consumption of respiratory substrates, ensuring energy supply, mitigating oxidative damage, and maintaining the integrity of the cell membrane, thereby enhancing the fruit’s resistance to stress (Wang et al., 2023).

Sucrose is an important form of sugar accumulation, closely related to fruit quality, energy metabolism, osmotic regulation, and stress resistance (Zhang et al., 2023b). Sucrose and hexose in cells can maintain a dynamic equilibrium through mutual conversion under the catalysis of multiple enzymes, which integrate sucrose into key sugar metabolism pathways like the pentose phosphate pathway and the TCA (Li et al., 2020b). It has been confirmed that the enzymes such as SS-s and SPS are instrumental in catalyzing the biosynthesis and storage of sucrose, whereas sucrose-consuming enzymes, like SS-c, AI, and NI, are implicated in the hydrolytic breakdown of sucrose into glucose and fructose (Cao et al., 2013). The results demonstrated that H_2_S treatment enhanced the enzymatic activities of SS-s and SPS in litchi fruit over the initial 48 hours, along with decrease in the activities of SS-c, AI, and NI. This may be caused by the improvement of the energy state in the fruit (Sun et al., 2020). The enzymatic modulation led to a higher sucrose synthesis, which is maintained throughout the intermediate and later phases of storage. Concurrent studies have also corroborated the notion that sustaining elevated sucrose levels can be advantageous in decelerating fruit senescence by securing a robust energy supply and strengthening antioxidant defenses in diverse fruit species, including peaches (Wang et al., 2023), apples (Chen et al., 2019), and raspberries (Shi et al., 2019). In light of these findings, the improvement of litchi browning by exogenous H_2_S treatment may be partly attributed to sucrose synthesis through regulating SPS, SS, AI and NI activities during storage.

Interestingly, as the essential substrates of respiratory metabolism, the content of fructose, glucose, and sucrose in the control fruit marginally increased after 48 h of storage, accompanied by the energy deficit of fruit. Past researches have demonstrated that under carbohydrate deficiency, alternative respiratory substrates such as proteins and lipids will participate in cellular respiration to regulate energy state (Araújo et al., 2011), which may lead to the maintenance or marginal increase of sugar pools in the plant tissue (King and Morris, 1994). The application of H_2_S has been observed to forestall the emergence of this metabolic shift, potentially attributable to the role of H_2_S in mitigating the depletion of sugar reserves in litchi. This observation suggests that H_2_S may exert a protective effect on the energy metabolism of litchi by preserving the endogenous sugar levels and reducing oxidative damage to the cell membrane, thereby preventing the premature decomposition of non-carbohydrate substrates in the respiratory process.

## 5 Conclusion

In conclusion, H_2_S treatment successfully reduced the postharvest pericarp browning in litchi fruit. H_2_S treatment enhanced energy state by modulating the expression of *LcAtpB*, *LcAOX1*, *LcUCP1*, *LcAAC1* and *LcSnRK2*, and the activities of H^+^-ATPase, Ca^2+^-ATPase, CCO and SDH. Moreover, H_2_S treatment reduced soluble sugar consumption in litchi fruit, promoted sucrose synthesis by controlling the activities of SPS, SS, AI and NI that are associated with sucrose metabolism, ensuring the energy supply and alleviating oxidative stress. These findings suggest that H_2_S treatment can delay the pericarp browning by enhancing the energy supply and antioxidant activity of litchi fruit. Further research is needed to determine the effects of H_2_S on other regulatory factors (e.g., signaling molecules and transcription factors) associated with sugar and energy metabolisms. Our results provide crucial insights into the mechanism of H_2_S inhibiting postharvest pericarp browning of litchi, providing a theoretical foundation for application of H_2_S in litchi preservation.

## Data Availability

The raw data supporting the conclusions of this article will be made available by the authors, without undue reservation.
